# The Disparities in Accessing Online Health Information and Health Care Utilization Among Older Americans

**DOI:** 10.1093/geroni/igaa057.1013

**Published:** 2020-12-16

**Authors:** Darren Liu, Taka Yamashita, Betty Burston, Jennifer Keene

**Affiliations:** 1 Des Moines University, West Des Moines, Iowa, United States; 2 University of Maryland, Baltimore, Maryland, United States; 3 University of Nevada Las Vegas, Las Vegas, Nevada, United States

## Abstract

Digital divide or unequal access to internet and other technology result in health disparities among older adults. This study examined possible disparities in accessing online health-related technology, and the associations between the online health-related technology use and health care utilization among older adults in the U.S. The data comprised a sample of 1,497 older adults aged 51 and older which were obtained from the 2014 Health and Retirement Study (HRS)’s supplemental module (Health behaviors). We used the survey-weighted negative binomial regression and binary logistic regression for the analyses. The results showed that older age, racial/ethnic minorities (e.g., Black and Hispanic vs. Whites & Other), being married, lower educational attainment, lower-income, being uninsured and reporting poorer health were associated with lower utilization of online health-management tools. In Addition, the use of online health-management tools was associated with 34% greater mean number of doctor visits (Incidence-Rate-Ratio = 1.34, S.E. = 0.10, p < 0.05) than non-use. However, the use of online health-management tools was not associated with hospitalization. Indeed, only health care needs - the self-rated health (Odds-Ratio = 0.58, S.E. = 0.18, p < 0.05) and the number of chronic conditions were associated hospitalizations (Odds-Ratio = 1.68, S.E. = 0.07, p < 0.05). More research is needed to clarify the purposes (e.g., prevention vs. treatment) and outcomes of health care service utilization within the context of health care technology use. Yet, it is important to proactively address digital divide as one of upstream strategies for reducing health and health care disparities.

